# Asthma diagnosis and treatment – 1014. Single center experience of 4 cases of diffuse panbronchiolitis clinically presented as treatment resistant ashtma

**DOI:** 10.1186/1939-4551-6-S1-P14

**Published:** 2013-04-23

**Authors:** Kyung Hee Park, Jae-Hyun Lee, Chein-Soo Hong, Jung-Won Park

**Affiliations:** 1 Division of Allergy and Immunology, Department of Internal Medicine, Yonsei University College of Medicine, South Korea

## Background

Diffuse panbronchiolitis (DPB) is a severe form of bronchiolitis affects the whole lung fields. Although its etiology is unknown, it can be well treated by macrolide administration. The incidence of DPB is high among Asian, especially Japanese and Korean. We experienced 4 DPB patients misdiagnosed to severe asthma or combined with asthma.

## Case 1

A 70-year old female came to our clinic complaining uncontrolled asthma for 10 years. Spirometry showed obstructive lesion (FEV1=94.9%, FEV1/FVC =71.08%) with 5% bronchodilator (BD) response. PC20 of methacholine was 3.8 mg/ml. Chest CT showed diffuse bronchial wall thickening with tree in bud sign. Treated with clarithromycin for 1 month, FEV_1_was improved from 64.9% to 84.2% without asthma medication.

## Case 2

A 52-year old male complained his uncontrolled asthma for 4years. He was diagnosed as severe asthma and treated at other hospital. His FEV1 was 1.74L (54.4%) and improved 20.6% after bronchodilator. CT scan showed diffuse bronchiolitis thus we adminster clarithromycin for 6 months. FEV_1_was improved from 54.4% to 95.3%. He could discontinue inhaledcorticosteroid and other asthma medications.

## Case 3

A 25-year old male with uncontrolled asthma. Initial FEV1 was 2.51 L (62.6%) and severe sputum eosinophilia. His CT scan shows diffuse bronchitis with tree bud sign and then treated with clarithromycin for 9 months. FEV_1_was improved from 62.6% to 88.4%. He reduced daily amount of inhaled corticosteroid.

## Case 4

A 60-year old female was visited to our clinic with severe asthma. Spirometry showed obstructive lesion (FEV1=42.9%, FEV1/FVC =73.37%) without BD response. CT scan shows diffuse bronchial wall thickening. FEV_1_was improved from 42.9% to 97.1 % after 6 months use of clarithromycin without any asthma medication.

## Conclusion

We report 4 cases of DPB were mistaken for severe asthma or combined with asthma. We suggested DPB must be considered as a differential diagnosis for treatment resistant asthmatics in Korea.

